# Clinical Diversity in Focal Congenital Hyperinsulinism in Infancy Correlates With Histological Heterogeneity of Islet Cell Lesions

**DOI:** 10.3389/fendo.2018.00619

**Published:** 2018-10-17

**Authors:** Ross J. Craigie, Maria Salomon-Estebanez, Daphne Yau, Bing Han, Walaa Mal, Melanie Newbould, Edmund Cheesman, Stefania Bitetti, Zainab Mohamed, Rakesh Sajjan, Raja Padidela, Mars Skae, Sarah Flanagan, Sian Ellard, Karen E. Cosgrove, Indraneel Banerjee, Mark J. Dunne

**Affiliations:** ^1^Paediatric Surgery, Royal Manchester Children's Hospital, University Manchester NHS Foundation Trust (MFT), Manchester, United Kingdom; ^2^Faculty of Biology, Medicine & Health, University of Manchester, Manchester, United Kingdom; ^3^Paediatric Endocrinology, Royal Manchester Children's Hospital, University Manchester NHS Foundation Trust (MFT), Manchester, United Kingdom; ^4^Paediatric Histopathology, Royal Manchester Children's Hospital, University Manchester NHS Foundation Trust (MFT), Manchester, United Kingdom; ^5^Nuclear Medicine, Royal Manchester Children's Hospital, University Manchester NHS Foundation Trust (MFT), Manchester, United Kingdom; ^6^Molecular Genetics, Royal Devon & Exeter NHS Foundation Trust, University of Exeter Medical School, Royal Devon & Exeter Hospital, Exeter, United Kingdom

**Keywords:** congenital hyperinsulinism, insulin, hypoglycaemia, islet, positron emission tomography, focal, pancreas, β-cell

## Abstract

**Background:** Congenital Hyperinsulinism (CHI) is an important cause of severe and persistent hypoglycaemia in infancy and childhood. The focal form (CHI-F) of CHI can be potentially cured by pancreatic lesionectomy. While diagnostic characteristics of CHI-F pancreatic histopathology are well-recognized, correlation with clinical phenotype has not been established.

**Aims:** We aimed to correlate the diversity in clinical profiles of patients with islet cell organization in CHI-F pancreatic tissue.

**Methods:** Clinical datasets were obtained from 25 patients with CHI-F due to *ABCC8/KCNJ11* mutations. ^18^F-DOPA PET-CT was used to localize focal lesions prior to surgery. Immunohistochemistry was used to support protein expression studies.

**Results:** In 28% (*n* = 7) of patient tissues focal lesions were amorphous and projected into adjoining normal pancreatic tissue without clear delineation from normal tissue. In these cases, severe hypoglycaemia was detected within, on average, 2.8 ± 0.8 (range 1–7) days following birth. By contrast, in 72% (*n* = 18) of tissues focal lesions were encapsulated within a defined matrix capsule. In this group, the onset of severe hypoglycaemia was generally delayed; on average 46.6 ± 14.3 (range 1–180) days following birth. For patients with encapsulated lesions and later-onset hypoglycaemia, we found that surgical procedures were curative and less complex.

**Conclusion:** CHI-F is associated with heterogeneity in the organization of focal lesions, which correlates well with clinical presentation and surgical outcomes.

## Introduction

The most common cause of persistent or recurrent hypoglycaemia in early childhood and infancy is hyperinsulinism. Congenital Hyperinsulinism in Infancy (CHI), a rare disorder, arises from inappropriate insulin release for the level of glycaemia. Despite advances in genetic testing and management strategies, CHI carries significant morbidity and mortality associated with hypoglycaemia-induced brain injury and adverse long-term neurological outcomes (1–6). The hypoglycaemia can be unresponsive to dietary and medical interventions with diazoxide, somatostatin analogs and other medications necessitating focal lesionectomy, partial or near-total pancreatectomy (7–10). The extent of surgery performed depends upon the developmental nature of the underlying genetic cause of disease, which is broadly classified as either focal or diffuse (11). Diffuse β-cell hypersecretion requires near total pancreatectomy, and invariably results in additional complications such as diabetes mellitus and/or exocrine pancreas insufficiency (12–14). Focal disease (CHI-F) on the other hand is caused by β-cell hyperfunction and hyperplasia, which is localized to a solitary functional lesion (15). As CHI-F can be surgically managed in many cases by excision of the lesion, pre-operative localization techniques have been used to guide surgical intervention toward the removal of disease tissue from non-affected parts of the pancreas. Localization techniques range from interventional investigations, such as pancreatic venous sampling (PVS) and combined selective pancreatic arterial calcium stimulation and hepatic venous sampling to fluorine-18 labeled fluoro-L-DOPA (^18^F-DOPA) positron emission tomography (PET) imaging. ^18^F-DOPA PET currently has the highest degree of specificity in diagnosing CHI-F and has largely replaced other investigations in the localization of focal domains (16–18).

Mutations in 11 genes (*ABCC8, KCNJ11, GCK, GLUD1, HADH, PGM1, HNF1a, HNF4a, INSR, FOXA2*, and *CACNA1D*) and the inappropriate expression of *SLC16A1* and *HK1* have been associated with CHI (19). Loss-of-function mutations in subunits of the pancreatic ATP-sensitive potassium (K_ATP_) channel, *ABCC8* and *KCNJ11*, account for more than 50% of CHI cases (19–21) and for all reported CHI-F cases. CHI-F is highly unusual in its pathogenesis as it involves the inheritance of a paternal germinal mutation (*ABCC8* or *KCNJ11*) and loss of the maternal imprinting of chromosome 11p15 allele—loss of heterozygosity (LOH). LOH is a somatic event restricted to a group of β-cells (15, 22). Amongst the genes lost from the maternal allele are *ABCC8/KCNJ11*, and several genes that normally result in balanced cell growth including *H19, IGF-II*, and *CDKN1C*. For CHI-F this combination of events leads to the expansion of a β-cell mass harboring defects in K_ATP_ channels within a localized domain (22). We know little about the triggers for maternal LOH during embryogenesis, and since the timing of this event is likely to be variable, it is reasonable to assume that this will lead to heterogeneity in the formation and organization of CHI-F lesions with variable clinical impact. Indeed, whilst there are several reports of the occurrence of unusual focal lesions in CHI patients, there has been no data to correlate structural aspects of lesions to the clinical profiles. Here, we report a series of 25 cases of CHI-F in which we were able to separate the organizational aspect of focal lesions into two categories that have a direct correlation to clinical datasets, surgical management, and outcomes.

## Subjects and methods

### Cohort

Our study is based upon 25 subjects with CHI-F originating from a single specialized treatment center for CHI using established diagnostic criteria (20). Twenty four patients were aged between 1 and 19 months at the time of surgery, Table 1. One subject was not diagnosed with CHI-F until after death at 4 months of age (5). Pancreatic surgery was performed for alleviation of sustained hypoglycaemia in patients with poor or inappropriate responses to medical therapy, Table 2. All pancreatic tissue for research was used in accordance with ethical approval, national codes of practice and informed consent.

**Table 1:** Characteristics of the CHI patient cohort.

**Code**	**Typing**	**Weight (Kg)**	**Age of onset (days)**	**Age at surgery (months)**	**PET-CT outcome**	**Surgery**	**Post-operative & Long-term outcome**
CHI-F1	2	4.2	7	1	**	Subtotal	Normoglycaemia. Learning difficulties. Visual impairment
CHI-F2	1	3.75	1	1	**	Subtotal	Hyperglycaemia. Diabetes
CHI-F3	1	4.8	1	2	**	Subtotal	Short-term Hypoglycaemia. Learning difficulties
CHI-F4	2	4.5	3	3	Focal uptake	Focal	Normoglycaemia. Autism
CHI-F5	2	4.5	120	19	Focal uptake	Focal	Short-term Hypoglycaemia. Healthy
CHI-F6	1	3.6	7	3	Focal uptake	Extended	Normoglycaemia. Healthy
CHI-F7	2	4.1	90	10	Focal uptake	Focal	Normoglycaemia. Healthy
CHI-F8	1	3.6	120	5	Focal uptake	Subtotal	Normoglycaemia. Healthy
CHI-F9	2	4.6	90	7	Focal uptake	Focal	Normoglycaemia. Healthy
CHI-F10	1	3.3	3	2	Heterogeneous	Extended	Normoglycaemia. Healthy
CHI-F11	2	2.9	1	3	Focal uptake	Focal	Normoglycaemia. Healthy
CHI-F12	2	6.5	1	3	Focal uptake	Focal	Normoglycaemia. Healthy
CHI-F13	2	3.5	3	4	Focal uptake	Focal	Normoglycaemia. Healthy
CHI-F14	1	4.34	4	2	Focal uptake	Extended	Hypoglycaemia. Medical treatment
CHI-F15	2	4.4	90	8	Focal uptake	Focal	Normoglycaemia. Developmental delay
CHI-F16	1	4.1	2	–	**	n/a	n/a
CHI-F17	2	1.9	90	5	Focal uptake	Focal	Normoglycaemia. Healthy
CHI-F18	2	3.6	150	8	Focal uptake	Focal	Normoglycaemia. Healthy
CHI-F19	2	3.9	6	2	Heterogeneous	Focal	Normoglycaemia. Feeding problems
CHI-F20	2	2.9	3	2	Focal uptake	Focal	Normoglycaemia. Healthy
CHI-F21	2	10.6	180	16	Focal uptake	Focal	Normoglycaemia. Healthy
CH1-F22	2	4.8	2	2	Focal uptake	Focal	Normoglycaemia. Healthy
CHI-F23	2	4.6	1	3	Focal uptake	Focal	Normoglycaemia. Healthy
CHI-F24	2	6.2	1	1.5	Focal uptake	Focal	Normoglycaemia. Healthy
CHI-F25	2	5.76	1	3.5	Focal uptake	Focal	Normoglycaemia. Feeding problems

All patients were treated for hypoglycaemia and classified as focal CHI based upon clinical characteristics, genotyping (Table 2), PET-CT diagnosis or pancreatic histology following surgery. In 24/25 cases, patients underwent surgery to alleviate hyperinsulinism. Surgery was defined as subtotal pancreatectomy (“Subtotal”), lesionectomy (“Focal”) and extended focal surgery (“Extended”). Details of the clinical outcomes—post-operative and long-term, are indicated. In all cases, histological profiles were used to classify the pathology as either Type 1 or Type 2 CHI-F. CHI-F16 surgery was not performed as the patient died. ^**^Not available.

**Table 2:** Medical treatment of the CHI patient cohort.

**Code**	**Glucose requirement (mg/kg/min)**	**Medical treatment**	**Response to medical treatment**
**TYPE 1 CASES**			
CHI-F2	20.4	Diazoxide	Partial
CHI-F3	20	Diazoxide & Octreotide	Diazoxide-responsive; Octreotide-partial
CHI-F6	15.6	Diazoxide & Octreotide	Diazoxide-responsive; Octreotide-partial
CHI-F8	18	Diazoxide & Octreotide	Diazoxide-unresponsive; Octreotide-partial
CHI-F10	20.8	Diazoxide & Octreotide	Diazoxide-unresponsive; Octreotide-responsive
CHI-F14	20	Diazoxide & Octreotide	Diazoxide-unresponsive; Octreotide-responsive
CHI-F16	None	Untreated	Untreated
**TYPE 2 CASES**			
CHI-F1	Not available	Diazoxide	Partial at high dose
CHI-F4	16	Diazoxide & Octreotide	Partial
CHI-F5	13	Diazoxide & Octreotide	Diazoxide-unresponsive; Octreotide-partial
CHI-F7	Not available	Diazoxide	Partial at high dose
CHI-F9	Not available	Diazoxide	Unresponsive
CHI-F11	Not available	Diazoxide & Octreotide	Diazoxide-unresponsive; Octreotide-responsive
CHI-F12	Not available	Diazoxide & Octreotide	Diazoxide-unresponsive; Octreotide-partial
CHI-F13	15	Diazoxide & Octreotide	Diazoxide-unresponsive; Octreotide-partial
CHI-F15	Not available	Diazoxide & Octreotide	Both partial
CHI-F17	Not available	Diazoxide & Octreotide	Diazoxide-unresponsive; Octreotide-responsive
CHI-F18	15	Diazoxide & Octreotide	Diazoxide-unresponsive; Octreotide-partial
CHI-F19	15.4	Diazoxide & Octreotide	Both unresponsive
CHI-F20	14.3	Diazoxide & Octreotide	Diazoxide-unresponsive; Octreotide-partial
CHI-F21	Not available	Diazoxide & Octreotide	Diazoxide-unresponsive; Octreotide-responsive
CHI-F22	20.8	Diazoxide & Octreotide	Both unresponsive
CHI-F23	13.9	Diazoxide & Octreotide	Diazoxide-unresponsive; Octreotide-responsive
CHI-F24	17.7	Diazoxide & Octreotide	Diazoxide-unresponsive; Octreotide-partial
CHI-F25	23	Diazoxide & Octreotide	Diazoxide-unresponsive; Octreotide-partial

*Summary of the medical treatment profiles of patients grouped by the type of histological lesion*.

### Tissue samples, histochemistry, and immunohistochemistry

Immunohistochemistry was performed as described previously on heat-mediated antigen retrieved 5 μm thick sections of tissue, which were fixed in 4% paraformaldehyde overnight and embedded in paraffin wax as previously described (23–26). All slides were then digitized as described previously (23–26).

### Statistical analysis

Unless otherwise stated data are presented as mean ± standard error and a one-way analysis of variance (ANOVA) was used to determine whether there were significant differences among the means of datasets.

## Results

### Heterogeneity in the structure of focal lesions

**Figure 1:**
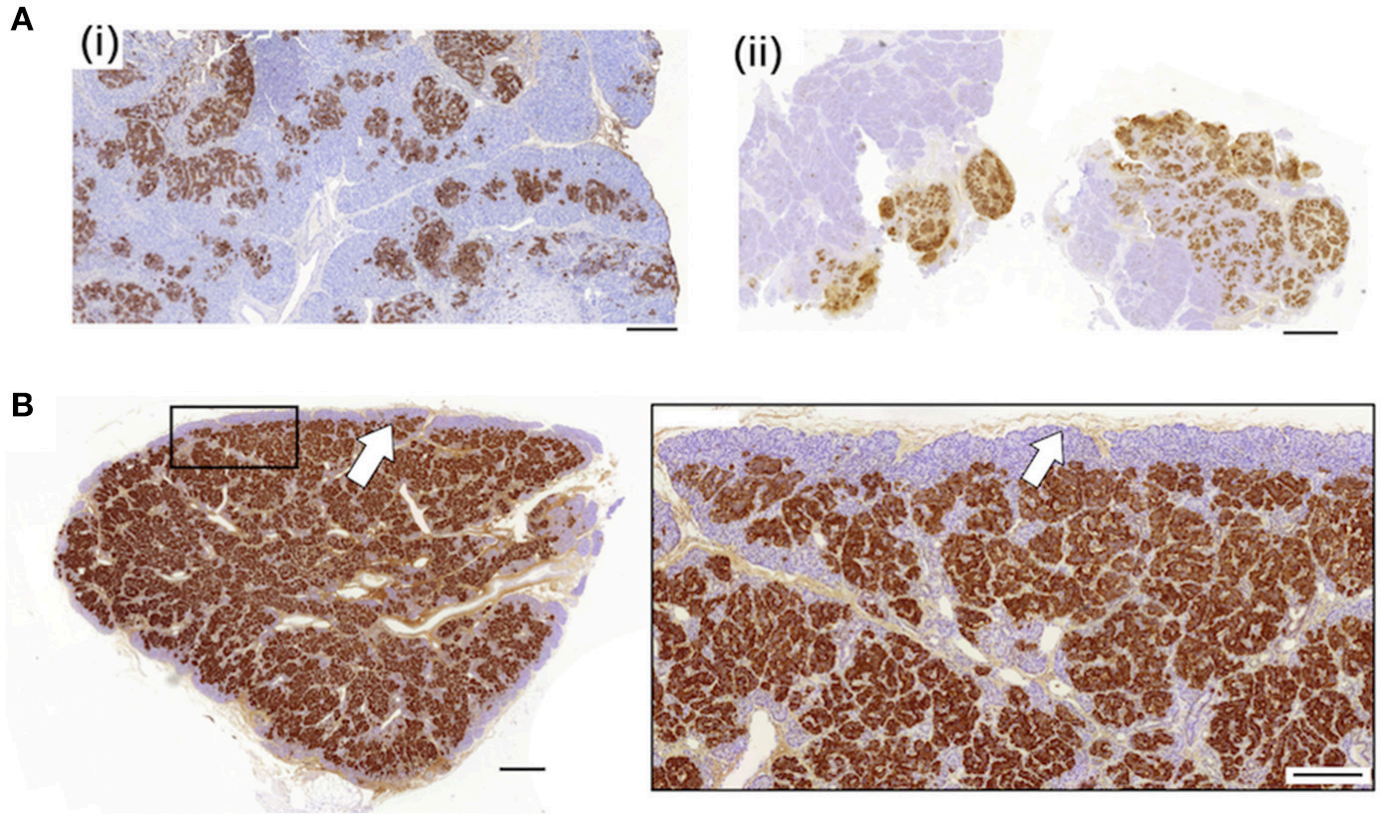
Diverse histopathology in focal CHI. Two discrete profiles of CHI-F are represented in this figure. Panel **(A)** shows typical images of Type 1 CHI-F tissue from two patients [CHI-F14 (i) and CHI-F3 (ii)]. The endocrine mass—insulin-positive cells, is poorly defined and not encapsulated. By contrast, in Type 2 tissue, Panel **(B)** (data obtained from CHI-F7), islet cell hyperplasia is highly localized and demarcated with a defined matrix capsule, arrow. Scales bars: Panel **(A)** (i) and (ii) 0.2 mm; Panel **(B)** (i) 0.5 mm, (ii) 0.2 mm.

**Figure 2:**
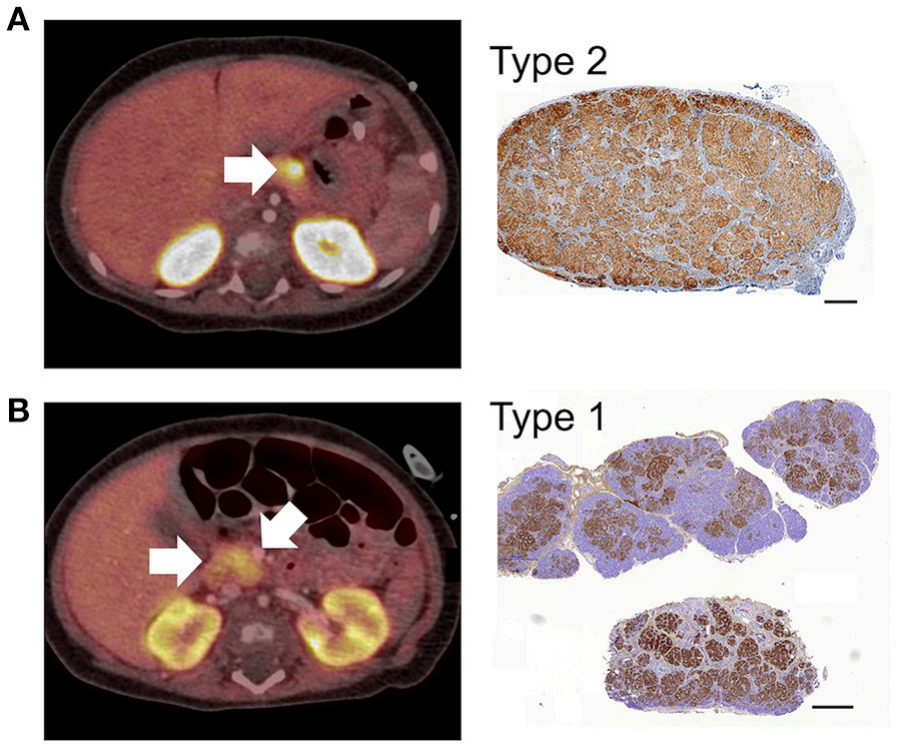
^18^F-DOPA PET-CT images and CHI-F heterogeneity. All panels show combined positron emission tomography (PET)/computed tomography (CT) scan images and the corresponding immunohistochemical images (Ins^+^) of focal lesions following surgery. In Panel **(A)**, the PET/CT scan reveals a discrete lesion with high uptake of tracer in the distal body of the pancreas. The corresponding histological data shows that the focal lesion was highly localized,—typical Type 2 lesion. Panel **(B)** shows a typical Type 1 lesion. The PET/CT scan reveals heterogeneous uptake of tracer with the highest SUV in the neck of pancreas (arrow). The corresponding histological data shows a heterogeneous lesion domain with expansion of islet cells into the healthy region of the pancreas. The data in **(A)** was obtained from patient CHI-F17 and **(B)** from CHI-F10. Scale bars 0.5 mm.

To investigate the relationship between clinical phenotype and islet lesions, we assessed the microscopic structures of focal lesions using tissues from patients following surgery. This revealed two broad types of lesions. First, islet tissues were found to be loosely connected within a focal area with extensions and organized clusters of islet cells within normal tissue, Figures 1A, 2A. In these cases (*n* = 7/25)—designated Type 1 lesions, the expansion of islet cell mass was not contained or encapsulated within a collagen matrix boundary that was continuous with the lesion. Second, in *n* = 18/25 cases we found that islet cells were localized to a discrete focal lesion and encapsulated by a defined extracellular matrix to support the confinement of cells, Figure 1B (see also Figure 2B). These lesions we designated Type 2, and we found no evidence of islet cells hyperplasia into the surrounding normal tissue. The size of the area occupied by the focal lesion was significantly larger in tissues with a poorly defined structure than those lesions with a defined organization 49.6 ± 13.6 mm^2^ (mean ± S.E.M., *n* = 7; range 6–103 mm^2^) vs. 23.2 ± 3 (*n* = 18; range 8–60.7 mm^2^), *p* < 0.02.

### Early- and late-onset hypoglycaemia in focal CHI patients

Using the tissue typing criteria of focal lesions we undertook a retrospective study of the clinical datasets of all 25 focal-CHI patients in our center, Table 1. Patients with Type 1 focal lesions had marked differences in the onset time of severe hypoglycaemia compared to patients with Type 2 focal lesions. On average, early-onset hypoglycaemia was detected 2.8 ± 0.8 days (*n* = 7, range 1–7 days) following birth compared to 56.1 ± 14.3 days (*n* = 18, range 1–180 days) in patients with Type 2 lesions. In this sub-group, patients were also younger at the time of surgery compared to late-onset patients, 2.5 ± 0.6 months (*n* = 6; 1 to 5 months) vs. 5.1 ± 1.1 months (*n* = 18; range 1 to 19 months). These differences were unrelated to birth-weight (a marker of intrauterine anabolic effects of insulin) 3.9 ± 0.2 kg (*n* = 7) vs. 4.4 ± 0.4 kg (*n* = 18), medical therapy (Table 2), genetic cause of disease (Table 3) or location of the lesion.

**Table 3:** Genotype characteristics of the CHI patient cohort.

**Code**	**Gene**	**Genotype**	**Protein**	**Location**	**Type**
**TYPE 1 CASES**					
CHI-F2	ABCC8	c.3600_3605del	p.Leu1201_Ser1202del	Exon 29	In-frame deletion
CHI-F3	ABCC8	c.1333-1013A >G/ None	p.?	Intron 8	Aberrant Splicing
CHI-F6	ABCC8	c.4287del/None	p.Val1430fs	Exon 35	Frameshift
CHI-F8	ABCC8	c.2524C>T/None	p.Arg842	Exon 21	Stop gain
CHI-F10	ABCC8	c.382G>A /None	p.Glu128Lys	Exon 3	Missense
CHI-F14	ABCC8	c.35C>A/None	p.Ser12	Exon 1	Stop gain
CHI-F16	Unknown	–	–	–	–
**TYPE 2 CASES**					
CHI-F1	KCNJ11	c.902G>A/None	p.Arg301His	n/a	Missense
CHI-F4	ABCC8	c.725del/None	p.Lys242fs	Exon 5	Frameshift
CHI-F5	KCNJ11	c.346T>C/None	p.Ser116Pro	n/a	Missense
CHI-F7	ABCC8	c.2995C>T/None	p.Arg999	Exon 25	Stop gain
CHI-F9	ABCC8	c.2116+1G>C/None	p.?	Intron 15	Aberrant Splicing
CHI-F11	ABCC8	c.3512del/None	p.Leu1171fs	Exon 28	Frameshift
CHI-F12	ABCC8	c.1879del/None	p.His627fs	Exon13	Frameshift
CHI-F13	ABCC8	c.946G>A /None	p.Gly316Arg	Exon 6	Missense
CHI-F15	ABCC8	c.1330C>T/None	p.Gln444	Exon 8	Stop gain
CHI-F17	ABCC8	c.1467+5G>A/None	p.?	Intron 9	Aberrant Splicing
CHI-F18	ABCC8	c.2509C>T/None	p.Arg837	Exon 21	Stop gain
CHI-F19	KCNJ11	c.286G>A/None	p.Ala96Thr	n/a	Missense
CHI-F20	ABCC8	c.2041-21G>A/None	p.?	Intron 14	Aberrant Splicing
CHI-F21	ABCC8	c.4310+1G>A/None	p.?	Intron 35	Aberrant Splicing
CHI-F22	ABCC8	c.1924-1G>T/None	p.?	Intron 13	Aberrant Splicing
CHI-F23	ABCC8	c.275G>A/None	p.Gly92Asp	Exon 2	Missense
CHI-F24	ABCC8	c.1879del/None	p.His627fs	Exon 13	Frameshift
CHI-F25	ABCC8	g.(17428434_17428336)_(17427000_17426289) del	p.?	Exon 26-27	Partial gene deletion

*Summary of the genetic profiles of patients grouped by the type of histological lesion. All patients were positive for gene defects in either ABCC8 or KCNJ11. Predicted protein structures have been indicated along with exon location and the type of mutation. Note that KCNJ11 is a single open reading frame. Five novel mutations were found in this cohort; CHI-F2, -F9, -F13, -F22, and -F25*.

In patients harboring discrete encapsulated focal lesions, a localized/focal uptake of ^18^F-DOPA in PET was diagnostic in 94% of cases (*n* = 16/17), Table 1 and Figure 2A. During surgery (*n* = 17), encapsulated lesions were visible, palpable (laparotomy), and easily dissected (laparoscopy), thereby making surgery less complicated. There were no post-operative complications in this group. Apart from one patient, normoglycaemia was achieved soon after lesionectomy. We found that 88% of children had no long-term adverse consequences (*n* = 15/17), Table 1.

For patients with poorly demarcated/unencapsulated focal lesions—Type 1, 75% of cases (*n* = 3/4) were also found to have a focal uptake pattern following PET-CT. Only one patient (CHI-F10) had a heterogeneous pattern of ^18^F-DOPA, Figure 2B. For these patients surgical procedures were more complex as only 33% (*n* = 2/6) of lesions were visible at the time of surgery and no lesion was palpable in open laparotomy. This led to a requirement for greater tissue dissection and more than one frozen section biopsy to find the anatomical location of the focal lesion. All patients who underwent surgery for Type 1 lesions (*n* = 6) required either an extended resection or a subtotal pancreatectomy to stabilize blood glucose levels (Table 1). Long-term outcomes were less favorable for these patients. Recurrent but transient hypoglycaemia was noted in three (50%) patients; both short- and long-term complications ranging from developmental delay, blindness, and hyperglycaemia, were also noted, Table 1.

## Discussion

Inappropriate insulin release from the CHI pancreas results from abnormalities in either all islets of Langerhans—diffuse disease, or from localized defects originating from a small focal lesion, CHI-F. The genetic basis of CHI-F has been widely reported, but the pathobiology of the lesion and impact upon the pancreas has not been thoroughly addressed. Further, whilst clinical heterogeneity in CHI-F has been reported (27, 28), the underlying associations or causes of this is unknown. In this study, we describe a direct correlation between the severity of focal CHI and the extent of lesion encapsulation. Islet tissue that is loosely organized and devoid of a matrix capsule—designated Type 1, causes early-onset hypoglycaemia, requiring complex surgery and suboptimal surgical outcomes. By contrast, later-onset disease with optimal outcome is directly correlated with marked islet cell encapsulation, designated Type 2. These observations may form the basis of a diagnostic process and are a step forward to understanding not only the pathobiology of the disease but also correlating histopathology with clinical phenotype.

CHI-F is a somatic-recessive endocrine disorder arising through a combination of events involving deregulation of imprinting in region Ch.11p15.5 and a somatic reduction to homozygosity of a paternal gene defect in either *ABCC8* or *KCNJ11*. At least 12 imprinted genes are known to be encoded by the Ch.11p15.5 imprinting domain and several are thought to be involved with β-cell overgrowth—*H19, P57KIP2*, and *IGF2*, in CHI-F (11, 15). Thus, at some stage during development and subsequent to islet cell formation, loss of maternal imprinting results in the inappropriate proliferation of β-cells harboring loss of function defects in K_ATP_ channel genes.

Normally, islets of Langerhans have a highly organized structure involving the spatial arrangement of endocrine cells, which are encapsulated and vascularized. The interface between islet and exocrine tissue is composed of an interstitial matrix and a basement membrane. Whilst the islet basement membrane acts as a functional barrier separating the exocrine and endocrine domains, the connective tissue/extracellular matrix (ECM) also serves a diverse number of functions (29–31). These include organizing the connections between endocrine cells, vascular endothelial cells, neural cells, and immune cells. These interactions have functional significance by facilitating the rapid exchange of oxygen, nutrients, metabolites, signaling hormones, and islet hormones (32, 33). In focal disease, not only does β-cell proliferation cause a marked increase in β-cell numbers, but it will also lead to a deregulation of the interactions between the ECM and islet cells. This is highly likely to lead to loss of functional integrity and may contribute to the pathobiology of disease.

There is little information on how timing of the loss of maternal imprinting impacts on the sequential organization of the islet cell architecture involving endocrine cellular proliferation, encapsulation and vascularization. Indeed, since loss of heterozygosity can occur at any time during embryogenesis, our data show that CHI-F is underpinned by heterogeneity in both structure and organization of the lesion domain. We found that in ~30% of patients, the organizational aspects of focal lesion were poor and the limits of the endocrine cell mass were difficult to define (5). This form of CHI-F has been alluded too by others in both large cohorts of patients or unusual case reports without reference to clinical severity of pathobiology (27, 28, 34–37). In this study, we found no correlation between the genetic cause of disease and the type of focal condition, but we did make the important observation that children with poorly formed lesions had much earlier onset of clinical symptoms than those children with discrete focal lesions. This may suggest a slower rate of endocrine cell and vascular proliferation in the latter group, but it seems more likely that this relates to differences in the manner of interactions with the ECM within the growing lesions. Because of the underlying architecture, it was easier to localize discrete lesions by ^18^F-Dopa PET-CT, undertake curative surgery and minimize long-term complications.

In summary, we have systematically examined the clinical profiles and the structure and organization of focal lesions in children with CHI-F. We found a degree of heterogeneity that was strongly correlated with disease severity, surgical management, and long-term clinical outcomes. The organization of islet cell structures is strongly determined by endocrine cells and their release of growth factors for vascular events. We therefore, conclude that the β-cell pathology in CHI-F extends beyond the loss of function defects in ATP-sensitive K^+^ channel genes and includes heterogeneity in interactions between islet cells and extracellular matrix organization.

## Ethics statement

This study was carried out in accordance with the recommendations of National Research Ethics Service (NRES) with written informed consent from all subjects. All subjects gave written informed consent in accordance with the Declaration of Helsinki. The protocol was approved by the North West Research Ethics Committee—Project Reference Number: 07/H1010/88.

## Author contributions

All authors contributed to the study and writing of the manuscript. RC, MSE, MS, KC, IB, and MD conceived the design of the study. MSE, DY, BH, WM, MN, EC, SB, ZM, RS, RP, SF, and SE collected data with the assistance of RC, IB, EC, and MD. Data were analyzed by IB and MD. The draft manuscript was prepared by MD and IB with critical review and agreement with all authors. The final manuscript was prepared by IB and MD.

### Conflict of interest statement

The authors declare that the research was conducted in the absence of any commercial or financial relationships that could be construed as a potential conflict of interest.
